# Data and performances evaluation of the SPIDIA-DNA Pan-European External Quality Assessment: 2nd SPIDIA-DNA laboratory report

**DOI:** 10.1016/j.dib.2016.01.062

**Published:** 2016-02-06

**Authors:** Francesca Malentacchi, Sara Pizzamiglio, Hady Ibrahim-Gawel, Mario Pazzagli, Paolo Verderio, Chiara Maura Ciniselli, Ralf Wyrich, Stefania Gelmini

**Affiliations:** a Department of Biomedical, Experimental and Clinical Sciences, University of Florence, Italy; b Fondazione IRCCS Istituto Nazionale dei Tumori, Milan, Italy; c QIAGEN GmbH, Hilden, Germany

## Abstract

Within the EU-SPIDIA project (www.spidia.eu), the quality parameters of blood genomic DNA were defined [SPIDIA-DNA: an External Quality Assessment for the pre-analytical phase of blood samples used for DNA-based analyses – [1]; Influence of pre-analytical procedures on genomic DNA integrity in blood samples: the SPIDIA experience – [2]; Combining qualitative and quantitative imaging evaluation for the assessment of genomic DNA integrity: the SPIDIA experience – [3]. DNA quality parameters were used to evaluate the laboratory performance within an External Quality Assessment (EQA) [Second SPIDIA-DNA External Quality Assessment (EQA): Influence of pre-analytical phase of blood samples on genomic DNA quality – [4]. These parameters included DNA purity and yield by UV spectrophotometric measurements, the presence of PCR interferences by Kineret software and genomic DNA integrity analysis by Pulsed Field Gel Electrophoresis.

Here we present the specific laboratory report of the 2nd SPIDIA-DNA EQA as an example of data and performances evaluation.

## **Specification table**

**Table:** 

**Subject area**	Molecular Biology
**More specific subject area**	External Quality Assessment for quality of genomic DNA from blood sample
**Type of data**	Table, text file, graph, figure
**How data was acquired**	Spectrophotometer (Nanodrop 1000 UV, Nanodrop), qPCR analysis (TaqMan RNaseP detection reagents, 7900HT Fast Real-Time PCR system, Applied Biosystems), Pulsed Field Gel Electrophoresis (CHEF DRII system, BioRad), Kineret software (Kineret Version 1.0.5, http://www.labonnet.com*)*
**Data format**	Analyzed
**Experimental factors**	The participants to the 2nd SPIDIA-DNA EQA received the same blood sample at 4 °C. After the genomic DNA (gDNA) isolation by their own procedure (within 3 days after sample arrival, storing blood at 4 °C), they sent back the extracted DNA at 4 °C at SPIDIA facility.
**Experimental features**	The defined DNA quality parameters (DNA purity and yield, presence of qPCR interferences and gDNA integrity) were evaluated according to an ad hoc statistical procedure by SPIDIA facility. A dedicated report was produced for each participant in which the performance related to each quality parameter was reported.
**Data source location**	University of Florence, Florence, Italy. Qiagen, Hilden, Germany. Labonnet Ltd. Company, Jordan Valley, Israel.
**Data accessibility**	Data is with this article.

**Value of the data**

• Identification of quality parameters for the evaluation of genomic DNA from blood.
• Analysis of the results of Pan European External Quality Assessment (EQA).
• Setting of a Report for the evaluation of laboratory performance for an External Quality Assessment.

## 1 Data

In order to inform the SPIDIA-DNA EQA participants on the quality of the extracted DNA from blood a dedicated report was realized [1], [4]. The report contains the evaluation of the performance of the specific laboratory and the overall distribution of the participants’ data for each gDNA quality parameter.

## 2 Experimental design, materials and methods

**Fig. 1:**
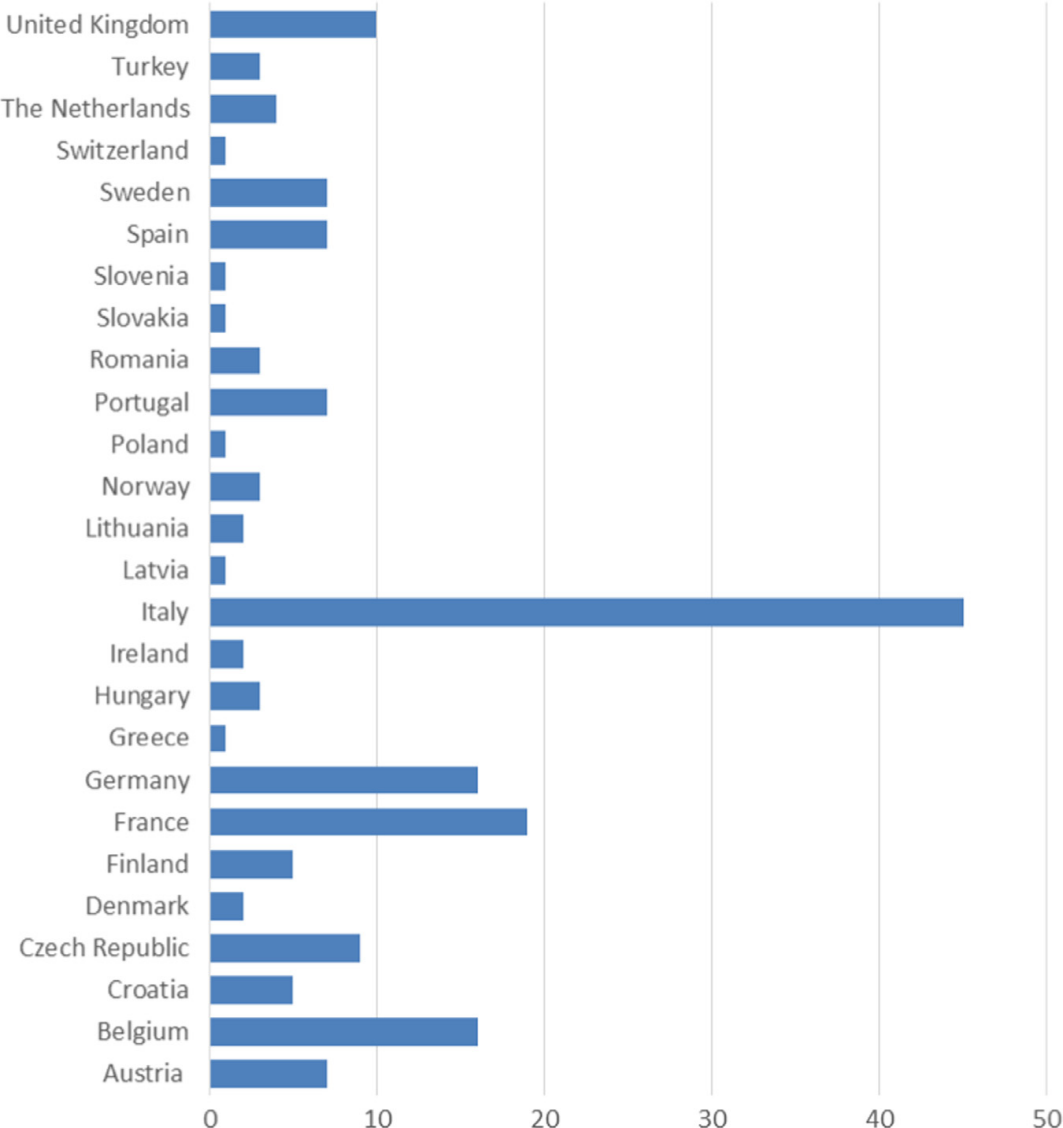
Distribution of the participating laboratories to the 2nd SPIDIA DNA EQA through Europe.

Briefly, 1.2 ml of blood from a single healthy donor was sent to 174 laboratories from 26 different European countries (Fig. 1). The participants extracted gDNA following their own procedure within 3 days from blood arrival, performed the spectrophotometric measurements and shipped back the sample at 4 °C. At SPIDIA facility, the defined DNA quality parameters (DNA purity and yield, gDNA integrity and of qPCR interferences) were evaluated and the performance of each laboratory was defined. A specific report (Appendix A, Supplementary material) was realized to give to the participants: (I) their own performance evaluation for each gDNA quality parameter; (II) the overall performance evaluation of the whole exercise and (III) the overall data distribution for each parameter.

### 2.1 gDNA purity and quantity evaluation

At SPIDIA facility, the absorbance was measured at 260 nm, 280 nm and 320 nm wavelengths by NanoDrop 1000 UV spectrophotometer (NanoDrop Technologies) [1], [4]. DNA purity (DNAb Spidia, Appendix A, Section A.3, right panel) was evaluated using 260 nm/280 nm ratio.

The DNA Quantity (DNAb Spidia, Appendix A, Section A.4, right panel) was evaluated as

Q (ng/µl blood)=[(260 nm)×50×dilution factor×elution volume]/extracted blood volume.

The specific laboratory DNA Purity (DNAb Lab, Appendix A, Section A.3, left panel) derived from the declaration in the Result Form.

The specific laboratory DNA Quantity (DNAb Lab, Appendix A, Section A.4, left panel) was computed by using the concentration (C), the extraction and elution volumes reported by the participants according to the following formula:

Q (ng/µl blood)=(C×elution volume)/extracted blood volume.

The performance of each laboratory in terms of DNA purity and quantity was evaluated by resorting to the two-step statistical procedure previously adopted [1], [4]. Briefly, we computed specific bootstrap centiles from the outlier-free distribution of DNA purity and yield derived from SPIDIA and laboratory data. Regarding DNA purity, the 2.5th and the 97.5th bootstrap centile were used to identify the lower and upper Action Limit (AL, blue lines) and the 10th and 90th bootstrap centile to identify the lower and the upper Warning Limit (WL, gray lines). For DNA quantity, we used the 5th and the 20th bootstrap centile to identify the one sided AL (blue line) and WL (gray line), respectively. According to these limits, the performance of each participant was classified as follows:

• Out of control: if the value exceeded the upper or the lower AL or if the value was below the one sided AL.
• Warning: if the value was between the upper AL and WL or between the lower AL and WL, or between the one sided WL and AL.
• In control: if the value was between the lower and the upper WL, or exceeded the one sided WL (Appendix A, Section A).

### 2.2 gDNA integrity

The gDNA integrity was analyzed by Pulsed Field Gel Electrophoresis (PFGE). 800 ng DNA were analyzed in a 1% agarose gel (Ultra Pure Agarose, Invitrogen), 0.5× TBE buffer (45 mM Tris, 45 mM Borate, 2.5 mM EDTA) and a CHEF DRII system (BioRad). Low Range PFG Marker (2.03–194 kb; New England Biolabs) was used as DNA size marker. Electrophoresis was performed for 16 h at 10 °C with 6 V/cm and a switch time of 1–12 s. The gel was stained for 30 min using 0.5 µg/ml ethidium bromide solution and destained for 1–2 h in distilled water. Documentation was performed using the EASY Win32 system (Herolab) [1], [4].

The images from PGFE were analyzed by ImageJ software and by a panel of expert. A dedicated algorithm, as described in Ciniselli at al. [3], was applied to combine the expert by-eye evaluation with the quantitative variables deriving from the ImageJ software. The final judgement classified the laboratory performance in one of the following 3 categories: low, intermediate high integrity (Appendix A, Section B).

### 2.3 qPCR interferences

The evaluation of qPCR interferences was made by Kineret software Version 1.0.5 (http://www.labonnet.com) as previously described [1], [4], [5].

Amplification curve of each sample was used to calculate the kineret distance (KD) from a reference curve. For SPIDIA DNA EQA analysis, all sample amplification curves were used as reference curve. The KD can be interpreted as a metric reflecting PCR interference due to unknown factors that adversely affects the PCR [5]. qPCR was performed using 1 μl of DNA and 11.5 μl of PCR mix containing 1× TaqMan RNaseP detection reagents (Applied Biosystems) and 1× Universal PCR Master Mix (Applied Biosystems) by 7900 HT Fast Real-Time PCR system (Applied Biosystems). Each sample was run in triplicate.

The performance was classified as follows:

• strong outlier (presence of interferences): value exceeding the 9.21 threshold;
• weak outlier (probably presence of some interferences): value between the 5.99 and the 9.21 threshold;
• in control (no interferences): value under the 5.99 threshold.

The message *missing* appeared when it was not possible to analyze the sample with the Kineret software (Appendix A, Section C).

### 2.4 Overall performance evaluation of the whole exercise

The participating laboratory performance for each quality parameter was summarized in a dedicated section (Appendix A, Section D), according to the traffic light color. When performance evaluation was not possible, a specific explanation was reported.

The performance was visually summarized by a table and a radar graph.

## Footnotes

Appendix A Supplementary data associated with this article can be found in the online version at doi:10.1016/j.dib.2016.01.062.
